# Clinical Significance of AFP and PIVKA-II Responses for Monitoring Treatment Outcomes and Predicting Prognosis in Patients with Hepatocellular Carcinoma

**DOI:** 10.1155/2013/310427

**Published:** 2013-12-29

**Authors:** Hana Park, Jun Yong Park

**Affiliations:** ^1^Department of Internal Medicine, CHA Bundang Medical Center, CHA University, Seongnam-si, Republic of Korea; ^2^Institute of Gastroenterology, CHA Bundang Medical Center, CHA University, Seongnam-si, Republic of Korea; ^3^Department of Internal Medicine, Yonsei University College of Medicine, 134 Sinchon-dong, Seodaemun-gu, Seoul, Republic of Korea; ^4^Institute of Gastroenterology, Yonsei University College of Medicine, Seoul, Republic of Korea; ^5^Yonsei Liver Cancer Special Clinic, Yonsei University College of Medicine, Seoul, Republic of Korea

## Abstract

*Aim*. Recently, the utility of tumor markers in the hepatocellular carcinoma (HCC) field has received a good deal of attention. Here, we review and summarize the results of studies on the roles played by the **α**-fetoprotein (AFP) and prothrombin induced by the absence of vitamin K or antagonist-II (PIVKA-II) responses in terms of the monitoring of outcomes and prediction of prognosis after various HCC treatments. *Methods*. Studies lodged in PUBMED and that satisfied our inclusion criteria were reviewed. *Results*. We reviewed 12 studies measuring both AFP and PIVKA-II responses in HCC patients treated in various ways. The results are presented by treatment modality. *Conclusion*. Measurement of AFP and PIVKA II marker levels before and after HCC treatment is clinically useful in monitoring of treatment outcomes and prognosis and in predicting recurrence and survival.

## 1. Introduction

Although treatments and surveillance have improved, hepatocellular carcinoma (HCC) remains difficult to cure, particularly when the disease is progressive. Because only limited treatment options are available, the prognosis is poor [1]. Thus, early diagnosis or detection of disease progression after treatment remains key to effective control of HCC. Imaging assessment remains the gold standard for evaluation of responses after various HCC treatments [2–4]. However, radiological analysis of HCC patients with vascular invasion or multiple lesions may not yield clear data on disease development, especially in cirrhotic patients. In addition, extensive desmoplastic and inflammatory reactions, and ischemic changes and tissue edema, develop after transarterial chemoembolization (TACE) or radiotherapy and may mask improvement in tumor size that is normally detectable by conventional imaging modalities, including radiology [5].

One possible way to deal with this limitation is via measurement of tumor markers, yielding information ancillary to imaging data. Such markers have been studied previously [6, 7]. Measurement of *α*-fetoprotein (AFP) level is simple and is already used widely for routine surveillance and noninvasive diagnosis of HCC and to evaluate prognosis and monitor recurrence following treatment [5, 6]. However, AFP serum levels can also be increased in patients with other nontumorous hepatic disorders, including acute and chronic hepatitis of any type, cirrhosis, and/or massive hepatic necrosis, and may reflect (general) hepatic inflammatory and regenerative activity [6, 8, 9]. However, serum prothrombin induced by the absence of vitamin K or antagonist-II (PIVKA-II) measurement not only more specifically differentiates HCC from other hepatic diseases [10, 11], but PIVKA-II levels are not also usually correlated with those of AFP [12, 13]. Thus, measures of PIVKA-II and AFP are independent. Although the AFP serum level reflects the intrahepatic tumor burden, assessment of serum PIVKA-II level reflects the extent of vascular invasion, including portal vein thrombosis and extrahepatic disease extension [14], and is regarded as complementary to serum AFP measurement. Thus, measurement of both PIVKA-II and AFP levels may yield useful information on treatment response and prognosis in HCC patients. Because the half-lives of the two serum markers are only a few weeks [15], changes in serum AFP and PIVKA-II levels before and after treatment may provide clinically useful information on both treatment outcome and prognosis. The clinical utility of simultaneous AFP and PIVKA-II measurement was first report by Aoyagi et al. in 1996 [16]. Since then, many studies have focused on use of a combination of the levels of these two markers to assess treatment response, to predict prognosis and indeed to diagnose HCC [17–23].

Multimodal treatment is mandatory in HCC patients, and the prognostic cutoff values, and predictive powers, of AFP and PIVKA-II levels will differ according to the chosen treatment modality. Therefore, we reviewed and summarized the results of studies on the utilities of AFP and PIVKA-II levels in monitoring of treatment outcomes and predicting prognosis.

## 2. Methods

### 2.1. Search Strategy

A computerized English-language search of PUBMED was performed in September 2013. Studies published at any time were included. After a preliminary search of the MeSH database, we used the terms “AFP and PIVKA-II,” “AFP and DCP,” “combination tumor markers,” “HCC,” “treatment response,” and “prognosis” to search titles and/or abstracts.

### 2.2. Study Eligibility and Critical Appraisal

We carefully reviewed all studies on AFP and PIVKA-II markers in the HCC context and selected studies (1) dealing with both AFP and PIVKA-II, (2) featuring measurement of both tumor markers before and after treatment of HCC, and, (3) focusing on the roles played by both tumor markers in assessing treatment outcomes or predicting prognosis and survival. We found 12 studies that met these criteria when investigating the utilities of various HCC treatment modalities (Table 1).

## 3. Results

### 3.1. Serum AFP and PIVKA-II Levels in Patients Who Underwent Curative Hepatic Resection to Treat HCC

Six studies measured both AFP and PIVKA-II levels in patients who underwent curative hepatic resection [20, 24–28].

#### 3.1.1. Monitoring Treatment Outcomes

Yamamoto et al. reported that the favorable predictive values of pretreatment AFP and PIVKA-II levels in terms of postoperative recurrence had AUROCs of 0.79 and 0.91, respectively [26]. The sensitivity and specificity of recurrence detection improved simultaneously when both AFP and PIVKA-II levels were measured (sensitivity, 66.7%; specificity 47.9%) compared to those obtained when AFP levels alone (sensitivity, 60.1%; specificity, 45.2%) or PIVKA-II levels alone (sensitivity, 62.9%; specificity, 47.9%) [20] were assessed. Patients with high pretreatment levels of AFP and PIVKA-II experienced a significantly higher incidence of tumor recurrence after curative treatment [20, 25], associated with the more unfavorable tumor characteristics of patients with higher levels of AFP and PIVKA-II [20, 21]. Chon et al. found that pretreatment AFP and PIVKA-II levels were significantly higher in patients with microscopic vessel invasion, or multiple tumors, compared to others [20]. The roles played by tumor markers in reflecting microscopic vessel invasion or tumor multiplicity can compensate for limitations of current prognostic systems.

The concern was whether changes in tumor marker levels after treatment would yield additional information on patients who underwent curative resection [20, 24, 25, 28]. Changes in tumor marker levels after curative hepatic resection provide information on both the pattern and probability of recurrence. In one study, high preoperative serum AFP and PIVKA-II levels were associated with early recurrence (within 6 months) after curative resection [26]. Such patients had higher preoperative AFP and PIVKA-II values than did those who developed recurrences >6 months after surgery. Also, patients experiencing extrahepatic recurrences had higher preoperative marker levels than did those with intrahepatic recurrences.

#### 3.1.2. Prediction of Survival

Serum AFP and PIVKA-II levels were also predictive of survival in many studies [20, 24–27]. Changes in tumor marker levels 3 months after operation significantly predicted HCC recurrence [20]. If marker levels did not fall, recurrence was likely [20, 24, 25]. Patients with high AFP and PIVKA-II levels after curative treatment experienced significantly poorer overall survival than those with normal marker levels [25]. In addition, not only high levels of markers per se, but also shorter doubling times of increases in marker levels were linked to significantly poorer disease-free and overall survival [27]. Thus, rapid elevation of marker levels reflects aggressive behavior of remnant tumors after curative treatment.

Upon multivariate analysis to evaluate the predictive values of marker levels in terms of survival, elevated serum levels of AFP and/or PIVKA-II both before and after surgery independently predicted disease-free or overall survival, as did tumor size, tumor number, and the existence of vascular invasion [20, 24, 25]. The numbers of markers elevated before operation and shorter doubling times of marker values were also predictive of survival [25, 27].

### 3.2. Serum AFP and PIVKA-II Levels in Patients with TACE

Two studies have examined the kinetics of both AFP and PIVKA-II levels in HCC patients treated via transarterial chemoembolization (TACE) [22, 29].

#### 3.2.1. Monitoring Treatment Outcomes

Radiological morphology after TACE is sometimes nonhomogenous and inconsistent because of irregular uptake of lipiodol and necrosis, evident on follow-up imaging, and this can compromise imaging-based measurements of tumor responses [32, 33]. Lee et al. and Park et al. evaluated the serum levels of AFP and PIVKA-II in efforts to overcome this limitation [22, 29]. Radiological responses were assessed using the modified Response Evaluation Criteria in Solid Tumors (mRECIST), and each patient was considered to show a complete response (CR), a partial response (PR), stable disease (SD), or progressive disease (PD), as described in previous reports [3]. When the percentage declines in tumor maker levels after treatment (from pretreatment levels) were evaluated, the reductions in both AFP and PIVKA-II levels in patients exhibiting a CR or PR were significantly greater than in those with SD or PD [29]. In addition, a strong association between the radiological response and serum AFP and PIVKA-II levels was evident [29]. However, such an association was questioned in another study [22]. The AFP serum level was significantly correlated with the radiological response, but the serum level of PIVKA-II was not.

#### 3.2.2. Prediction of Survival

Park et al. found significant differences in median overall survival times between tumor marker responders and nonresponders [29]. Upon multivariate analysis, the PIVKA-II and AFP responses were significant indicators of overall survival independent of host, tumor, and serological factors, when pretreatment values were compared with those 3 and 6 months after treatment. Lee et al. found that pretreatment AFP levels independently predicted progression-free survival, but pretreatment PIVKA-II levels did not. In terms of overall prediction of survival, the pretreatment PIVKA-II level, the presence of cirrhosis, the tumor number, and the AFP response were all independent predictors [22]. The cited authors performed a subanalysis to determine whether a combination of the AFP and PIVKA-II responses would improve the prognostic value of either alone. After stratifying patients with AFP and/or PIVKA-II responses into combined tumor marker responders, and those without AFP and PIVKA-II responses into combined tumor marker nonresponders, overall survival was significantly longer in the former than the latter group (39.0 versus 21.5 months; log-rank test, *P* = 0.011). In addition, the combined tumor marker response was an independent predictor of overall survival, together with tumor size and the presence of cirrhosis. However, in terms of prediction of progression-free survival, no difference was evident between the two groups. Thus, the combined tumor marker response did not independently predict progression-free survival upon multivariate analysis.

### 3.3. Serum AFP and PIVKA-II Levels Patients Receiving HAIC or CCRT to Treat HCC

Two studies examined the kinetics of both AFP and PIVKA-II levels in HCC patients undergoing hepatic artery infusional chemotherapy (HAIC) or concurrent chemoradiation therapy (CCRT) [14, 23].

#### 3.3.1. Monitoring Treatment Outcomes

Lee et al. evaluated the clinical utilities of AFP and PIVKA-II levels as predictors of treatment outcomes in patients with advanced HCC receiving HAIC (*n* = 60) or CCRT (*n* = 67) [23]. In patients who underwent HAIC, the overall response (both CR and PR, according to WHO criteria) was significantly higher in AFP responders than nonresponders (36.0% versus 8.6%, *P* = 0.009) and also in PIVKA-II responders than nonresponders (50.0% versus 2.5%, *P* < 0.001). However, no difference in disease control rate (the total of CR, PR, and SD, according to WHO criteria) between AFP or PIVKA-II responders and nonresponders was evident. In patients who underwent CCRT, only the overall response rate of PIVKA-II responders was significantly better than that of PIVKA-II nonresponders (42.2% versus 13.6%, *P* = 0.019); neither the overall response nor the disease control rate of AFP responders differed from those of AFP nonresponders. Another study on the clinical utilities of AFP and PIVKA-II levels in patients undergoing CCRT (*n* = 111) focused on whether a combination of AFP and PIVKA-II marker levels could be used to subdivide patients into prognostic groups [14]. Four groups were defined: A↓P↓ [AFP response (+) and PIVKA-II response (+)]; A↓P↑ [AFP response (+) and PIVKA-II response (−)]; A↑P↓ [AFP response (−) and PIVKA-II (+)]; A↑P↑ [AFP response (−) and PIVKA-II (−)]. Not only the overall response but also the disease control rate was the best in the A↓P↓ group, followed (in order) by the A↓P↑, A↑P↓, and A↑P↑ groups. Notably, this study showed that treatment outcome and prognosis differed significantly among patients varying in the PIVKA-II response, even when patients exhibited an AFP response. In addition, a combination of the responses of both tumor markers predicted the pattern of disease progression, extrahepatic versus intrahepatic. Extrahepatic disease occurred more frequently in the A↓P↑ group and intrahepatic disease more frequently in the A↑P↓ group (50.0% versus 28.6% for extrahepatic disease; 50.0 versus 71.4% for intrahepatic disease, respectively; *P* = 0.001). This is because the serum AFP level reflects the tumor burden, whereas the serum PIVKA-II level reflects the extent of vascular invasion (portal vein thrombosis and extrahepatic disease extension) [34].

#### 3.3.2. Prediction of Survival

In patients who underwent HAIC, AFP responders experienced significantly better overall survival than did AFP nonresponders (17.3 versus 6.4 months, *P* < 0.001), whereas the survival of PIVKA-II responders did not differ from that of PIVKA-II nonresponders [23]. Similar results were seen in patients who underwent CCRT. The overall survival of AFP responders was significantly longer than that of AFP nonresponders (17.6 versus 8.7 months, *P* = 0.014), but PIVKA-II responders and nonresponders did not significantly differ in this context. Rather, PIVKA-II responders among CCRT-treated patients showed significantly better progression-free survival than did nonresponders (9.2 versus 3.1 months, *P* < 0.001). Multivariate analysis revealed that the AFP response was independently predictive of overall survival in patients treated with HAIC or CCRT, whereas the PIVKA-II response predicted only progression-free survival in patients treated with CCRT. Park et al. found that the prognoses of AFP responders could be further divided in terms of whether such patients were also PIVKA-II responders [14]. Patients in the A↓P↓ group had significantly longer progression-free and overall survival than did those of the A↓P↑ group (16.2 versus 5.1 months, *P* = 0.009; 26.3 versus 7.3 months, *P* = 0.017, resp.) [14]. In addition, of patients who showed a discordant tumor marker response (A↓P↑ or A↑P↓), those of the A↑P↓ group who were AFP nonresponders had better progression-free and overall survival than did A↓P↑ patients (10.1 versus 5.1 months, *P* = 0.038; 22.4 versus 7.3 months, *P* = 0.038, resp.). The predictive power (in terms of survival) of the two combined tumor markers was better than that of AFP alone, and comparable to that of the radiological response, according to the mRECIST criteria.

### 3.4. Serum AFP and PIVKA-II Levels in Patients Given Sorafenib to Treat HCC

Two studies investigated the kinetics of both AFP and PIVKA-II after administration of sorafenib to patients with advanced HCC [30, 31].

#### 3.4.1. Monitoring Treatment Outcomes

Kuzuya et al. [30] measured tumor marker ratios (the concentrations of tumor markers 2 and 4 weeks after treatment, divided by the values before treatment). At 2 weeks, the AFP (but not the PIVKA-II) ratio was significantly higher in patients with PD than in those with PR or SD. At 4 weeks, both ratios were significantly higher in patients with PD than in those with PR or SD. The median AFP level did not change by either 2 or 4 weeks after commencement of sorafenib in the PR + SD group, but a significant increase was evident in the PD group. Similarly, Nakazawa et al. found an early increase in AFP level (more than 20% that of the baseline value) within 4 weeks after commencement of sorafenib in PD patients [31]. However, median PIVKA-II levels did not fall after commencement of sorafenib, even in the PR + SD group, in two studies [30, 31]. Rather, the median PIVKA-II levels at both 2 weeks and 4 weeks increased significantly over baseline in both the PR + SD and PD groups [30]. Such elevation of PIVKA-II levels even in patients who are responding well had been reported in previous studies [35, 36]. One possible explanation is that sorafenib-mediated inhibition of angiogenesis rendered tumor cells hypoxic, increasing PIVKA-II production [30, 36]. Therefore, the elevated PIVKA-II levels seen after administration of sorafenib may indicate not only tumor progression but also tumor responsiveness, and caution must be exercised when interpreting changes in AFP and PIVKA-II levels together.

#### 3.4.2. Prediction of Survival

Kuzuya et al. assessed the cumulative time to progression (using the RECIST criteria) and cumulative overall survival after dividing patients into two groups: those with low and high tumor marker ratios 4 weeks after treatment [30]. First, the median time to progression was significantly longer in the low than the high AFP ratio group (3.5 versus 2.1 months, *P* = 0.021), and the median overall survival tended to be higher in the former than the latter group, but the difference was not statistically significant (9.3 versus 5.1 months, *P* = 0.089). In terms of PIVKA-II levels, no significant difference in either cumulative time to progression or overall survival was evident between the low and high PIVKA-II ratio groups. Nakazawa et al. found that pretreatment PIVKA-II levels over 1,000 mAU/mL and an early increase in AFP level were independent predictors of poor overall survival. Further, an early rise in AFP level was the only independent predictor of poor progression-free survival [31].

## 4. Summary and Perspectives

The clinical utilities of tumor markers of HCC remain controversial. The potential roles played by tumor markers tend to be underrated in western reports, being considered of greater value in eastern settings. Recently, a combination of AFP and PIVKA-II levels has been recommended for diagnosis of HCC malignancy in Japan [17]. Most prior work on tumor markers focused on their possible diagnostic utility. However, as we have shown, measurement of marker levels both before and after HCC treatment is clinically valuable to monitor treatment outcomes (in combination with radiological analysis) and to predict prognosis, recurrence, and survival. Serum biomarkers demonstrate great potential for use in monitoring therapeutic effects and for predicting outcomes early in HCC treatment. Earlier studies used different “normal” values of AFP and PIVKA-II levels and variously defined measures of tumor response. It is necessary to standardize these measures during future evaluation of the importance of tumor markers in patients treated for HCC. In addition, HCC patient subdivision into groups defined by simultaneous consideration of AFP and PIVKA-II levels may yield interesting results.

In conclusion, measurement of a combination of tumor markers before and after HCC treatment is clinically valuable in terms of monitoring treatment outcomes (together with radiological analysis), and to predict prognosis recurrence, and survival.

## Figures and Tables

**Table 1:** Studies regarding both AFP and PIVKA-II in the patients who underwent various treatment modalities for HCC.

Author	Year	Number of patients	Treatment modality	Tumor marker	Cutoff value of markers	Definition of tumor marker response (change from baseline)
Toyoda et al. [24]	2012	173	Curative resection	AFP AFP-L3 PIVKA-II	20 ng/dL 5% 40 mAU/mL	—

Chon et al. [20]	2012	267	Curative resection	AFP PIVKA-II	20 ng/dL 40 mAU/mL	—

Nanashima et al. [25]	2011	470	Curative resection	AFP PIVKA-II	20 ng/mL, 200 ng/mL^†^ 40 mAU/mL, 400 mAU/mL^†^	—

Yamamoto et al. [26]	2009	714	Curative resection	AFP PIVKA-II	20 ng/mL 40 mAU/mL	—

Masuda et al. [27]	2010	210	Curative resection	AFP PIVKA-II	20 ng/mL 40 mAU/mL	—

Nanashima et al. [28]	2006	63	Curative resection	AFP PIVKA-II	20 ng/mL 40 mAU/mL	—

Lee et al. [22]	2013	115	TACE	AFP PIVKA-II	20 ng/mL 40 mAU/mL	≥50% reduction

Park et al. [29]	2012	327	TACE	AFP PIVKA-II	10 ng/mL 40 mAU/mL	≥50% reduction

Lee et al. [23]	2012	60 67	HAIC CCRT	AFP PIVKA-II	20 ng/mL 20 mAU/mL	≥20% reduction

Park et al. [14]	2013	111	CCRT	AFP PIVKA-II	200 ng/mL 60 mAU/mL	≥50% reduction

Kuzuya et al. [30]	2011	48	Sorafenib	AFP PIVKA-II	—^‡^	—

Nakazawa et al. [31]	2013	59	Sorafenib	AFP PIVKA-II	10 ng/mL 40 mAU/mL	≥20% increase Twofold increase

^†^Patients were divided into 3 groups with low and high cutoff values of tumor markers in this study.

^‡^Tumor marker ratio was evaluated in this study.

## Abbreviations

HCC: Hepatocellular carcinoma
AFP: *α*-Fetoprotein
PIVKA-II: Prothrombin induced by the absence of vitamin K or antagonist-II
TACE: Transarterial chemoembolization
AUROC: Areas under the receiver operating characteristics
mRECIST: Modified Response Evaluation Criteria in Solid Tumors
HAIC: Hepatic artery infusional chemotherapy
CCRT: Concurrent chemoradiation therapy.
